# Numerical characterization of noisy fluctuations in two different types of stochastic differential equation models of neural signaling

**DOI:** 10.1186/1471-2202-16-S1-P147

**Published:** 2015-12-18

**Authors:** Tiina Manninen, Jukka Intosalmi, Keijo Ruohonen, Marja-Leena Linne

**Affiliations:** 1 Department of Signal Processing, Tampere University of Technology, Tampere, Finland; 2 Department of Computer Science, Aalto University, Espoo, Finland; 3 Department of Mathematics, Tampere University of Technology, Tampere, Finland

## 

Stochastic modeling plays an essential role in the study of noise and random fluctuations in biochemical signaling of neural systems. Here, we study numerically noisy fluctuations produced by two different stochastic differential equation models, the chemical Langevin equation (CLE) model [1] and the rate constant stochastic differential equation (rcSDE) model [2], using a biologically realistic neuronal protein kinase C activation pathway [3] as a case study. We compare the CLE and rcSDE models by studying the noise power levels in different system volumes and with different model parameters. In this context, we also assess the problem of negative concentrations by computing the average number of state vectors containing negative concentrations within a single simulation run. Based on the information obtained from the examination of noise power levels, we then choose appropriate model parameters for the rcSDE model and the corresponding system volume for the CLE model. Using these parameters and the volume, we finally simulate the models and compare the results using multiresolution analysis.

In the simulations we present here, the rcSDE model is capable of producing the same level of the average power of noise with a notably smaller amount of negative concentrations than the CLE model. This difference is considerable and it suggests that in certain simulation settings, it might be reasonable to use rcSDE model as an approximate simulation approach if it is feasible to use the slightly different interpretation of the nature of noise. A more detailed multiresolution analysis reveals that the resulting noise processes differ at the higher frequencies. In many studies these differences at high frequencies are not of interest as neural biochemical systems often produce relatively stronger fluctuations at low frequencies. In some cases, however, noise might be important for the system function and, in such situations, it is crucial to make sure that the noise is modeled in an appropriate way. Whether the appropriate way is the CLE, the rcSDE model, or some other model is dictated by the application. Different models are based on different premises and it is modeler's task to pick up an appropriate model. We conclude that introduction of techniques from the field of signal processing may help assessing the role of noise in neural systems and what type of model to use. This study shows how the orthogonal wavelet representation or similar multiscale decompositions can be successfully applied to the study of noise in biochemical processes of neural systems.

## References

[B1] Gillespie DT The chemical Langevin equation J Chem Phys 2000 113 1 297 306

[B2] Manninen T Linne M-L Ruohonen K Developing Ito stochastic differential equation models for neuronal signal transduction pathways Comput Biol Chem 2006 30 4 280 291 16880117 10.1016/j.compbiolchem.2006.04.002

[B3] Bhalla US and Iyengar R Emergent properties of networks of biological signaling pathways Science 1999 283 5400 381 387 9888852 10.1126/science.283.5400.381

